# Minimally invasive surgical treatment on delayed uretero-vaginal fistula

**DOI:** 10.1186/s12894-018-0410-z

**Published:** 2018-10-29

**Authors:** Xinying Li, Ping Wang, Yili Liu, Chunlai Liu

**Affiliations:** Department of Urology, The Fourth Hospital of China Medical University, 4 Chongshan Road, Shenyang, 110032 China

**Keywords:** Ureter injury, Minimally invasive surgical, Endoscopy, Percutaneous nephroscopy, Ureterovaginal fistula

## Abstract

**Objective:**

To evaluate the procedure of endoscopic surgery for ureterovaginal fistula (UVF) and its clinical efficacy.

**Materials and methods:**

A retrospective analysis of 46 patients needing treatment for UVF with endourology technology was conducted (all patients had unilateral ureteric injury, 27 left and 19 right). Transurethral retrograde ureteric stenting or realignment retrograde/antegrade approach stenting was used to treat the fistula, and the relation between treatment and prognosis was analyzed.

**Results:**

One case failed, the patient undergoing percutaneous nephrostomy instead. Success was achieved in 45 cases, and urinary leakage was stopped 48 h after surgery. Of the 45 patients operated on, 16 had their double-J stents removed after 3–6 months, and 29 needed replacement every 6–12 months. In a postoperative follow-up of 6–36 months, 10 patients had recurrent stenosis needing ureteroscopic endoureterotomy or reexpansion with a balloon. No other complications occurred.

**Conclusions:**

Endoscopic surgery is an effective technology in the treatment of UVF, with the advantages of being effective, reliable, less invasive, and readily accepted by patients.

## Introduction

A ureterovaginal fistula (UVF) is an abnormal channel between the ureter and vagina, which is a severely disabling complication resulting in incontinence, infection, and discomfort; it is often diagnosed postoperatively [1]. The incidence of UVF has been increasing due to the growing use of the laparoscopic surgical technique [2]. Traditionally, a laparotomy or laparoscopic surgery has been used to repair the delayed UVF [3, 4]; however, the serious local inflammatory response has slowed surgical repair. In recent years, minimally invasive treatment has been widely used for this disease. We have retrospectively analyzed 46 patients who were treated with minimally invasive treatment of UVF at our hospital from 2006 to 2016. In this review, we discuss the treatments to repair UVF, the required techniques, and postoperative recommendations.

## Patients and methods

The mean age of selected patients suffering from ureterovaginal fistula was 42.5 years (range, 34–53). They all had unilateral ureteric injury (27 left and 19 right). All patients who were followed had delayed UVF after laparoscopic total hysterectomy (56.5%), laparoscopic radical resection of cervical cancer (34.8%), and laparotomy cervical cancer resection procedures (8.6%). The injuries were diagnosed in a median 3.7 days (range, 24 h to 42 days) after the primary procedure, when they were typically discovered having a urine leak from the vagina. The lesion-side ureters of these patients were opened; in the retroperitoneum, extravasation of contrast medium from the computed tomography urogram (CTU) of the distal ureter was revealed. The CTU revealed 35 patients with delayed kidney development lateral to the injuries. In the other 11 patients, renal imaging was normal. Ultrasound showed all of them to have hydronephrosis—19 mild and 27 moderate. A routine chemistry examination of the vaginal leakage revealed that urea and creatinine levels were close to normal urine. Flexible cystoscopy showed that the bladder mucosa was normal and that there was no blue-tinged urine extravasation in the vagina after injecting indigo carmine or methylene blue in the bladder to help rule out vesicovaginal fistula.

### Treatment

#### Ureteroscopy

Under continuous epidural or general anesthesia, all 46 patients were placed in the flank-reclining, split-leg position for simultaneous antegrade and retrograde ureteroscopy. We inserted an F16 ureteroscope in the bladder through the urethra. First, we made a ureteroscopic observation to again rule out vesicovaginal fistula. Then, a Zebra urological guide wire, carrying a ureteral scope, was inserted in the injured side of the ureter as far as the injury site. A Holmium laser was used when the cavity was too narrow or the surgery suture was met. Forty-six patients having a definitive diagnosis of delayed UVF were classified according to the lesion’s description, as follows:

- Class 1: The ureteric injuries were only fistulas, the ureteral mucosa was continuous, and the Zebra urological guide wire could be uplinked into the renal pelvis along the ureteral mucosa (34 cases).
- Class 2: More than half the diameter of the ureter was lacerated, a segment of the ureteral wall was a coloboma or collapsed, and the Zebra guide wire would not pass through the injury (9 cases).
- Class 3: The ureters were completely avulsed and the lacerated ends were filled in by the adjacent tissue (2 cases).
- Class 4: The injured ureter was completely atretic (1 case).

#### Ureteric stent placement

For Classes 1, two or three double-J (D-J) stents were indwelled over the injury through the Zebra guide wire, which uplinked into the renal pelvis along the ureter’s mucous membrane. The stents were confirmed to be in their appropriate positions during the operation with ultrasonography.

#### Endoscopic realignment for treatment [5]

The ureters of Class 2 were injured so seriously that the guide wire could hardly pass through the damaged region to reach the pelvis by retrograde ureteroscopy. Percutaneous nephroscopy had to be used to admit a flexible ureteroscope into the injury and insert the guide wire into the retroperitoneal space. A rigid ureteroscope was inserted retrogradely through the distal ureter to the injury. Then, a double ureteroscopic joint exploration and realignment was accomplished.

The guide wire was detected by the rigid ureteroscope, which was seized by the tip with an endoscopic grasping forceps and pulled out from urethra (using the foreign-body clamp in the ureteroscope to clip the guide wire outside the body through the diseased side of the distal ureter, bladder, and urethra), placing three D-J stents along the guide wire and over the fistula.

#### Treatment of ureteral occlusion

The ureters of Classes 3 and 4 were in complete occlusion, with the ureteroscope and guide wire unable to open the occluded portion. Therefore, a “cut-to-the-light” technique was employed [6], with the ureteral segments being aligned via ultrasonographic and endoscopic control. The room light was dimmed and the rigid ureteroscope’s light was turned off. Using the light source of the flexible ureteroscope that was inserted through the nephrostomy as a guide, we used the Holmium laser to restore ureteral continuity. A guide wire was indwelled across the area and uplinked to the renal pelvis, then three D-J stents were placed along the guide wire.

#### Treatment of secondary ureteric stricture

Ten patients had recurrent stenosis, which needed ureteroscopic endoureterotomy. First, we made an adequate longitudinal endoluminal incision of the strictured segment of the ureter and vaporized the scars until the periureteral fat was seen. Then, we indwelled three D-J stents for at least 6 weeks. We repeated the internal urethrectomy and replaced the stents after 6–12 months if the narrowing reappeared.

## Results

Results of the surgical treatment are shown in Table 1 and Table 2. Urine leakage decreased significantly and no obvious leakage became evident within 48 h postoperatively.

**Table 1:** Results list

	Age-bracket(y)	Side	Extent of injury	Initial operation	Time of recognition(days)	Operative time(min)	Hospital time(days)	Subsequent procedure
1	40–45	R	Class 1	a	8	30	3	III
2	35–40	L	Class 1	b	3	40	3	I
3	40–45	L	Class 1	b	6	40	3	I
4	40–45	L	Class 1	a	5	35	5	III
5	35–40	R	Class 1	b	2	30	3	I
6	45–50	R	Class 1	a	3	30	5	III
7	40–45	R	Class 1	c	4	35	4	III
8	40–45	L	Class 1	b	7	30	4	I&II
9	40–45	L	Class 1	b	2	30	3	III
10	45–50	R	Class 1	a	1	25	3	I
11	40–45	L	Class 1	a	2	25	4	I
12	25–35	L	Class 1	a	5	25	4	I&II
13	35–40	R	Class 1	a	2	35	4	III
14	40–45	L	Class 1	a	3	30	4	I
15	50–55	L	Class 1	a	2	40	5	I&II
16	40–45	L	Class 1	a	2	40	4	I
17	45–50	R	Class 1	b	1	60	3	I
18	35–40	L	Class 1	a	2	50	5	III
19	40–45	L	Class 1	b	8	50	3	III
20	35–40	L	Class 1	a	1	40	3	III
21	40–45	R	Class 1	a	5	40	4	I
22	25–35	R	Class 1	b	6	30	4	I&II
23	50–55	R	Class 1	a	2	30	3	III
24	50–55	R	Class 1	a	4	25	4	III
25	40–45	L	Class 1	b	5	30	4	I
26	45–50	L	Class 1	a	10	40	4	III
27	50–55	R	Class 1	b	4	40	4	I
28	40–45	L	Class 1	b	6	35	3	III
29	40–45	L	Class 1	b	1	30	5	III
30	40–45	R	Class 1	a	3	40	5	III
31	25–35	L	Class 1	a	3	35	4	I
32	35–40	L	Class 1	a	3	40	3	I
33	35–40	R	Class 1	b	1	40	3	III
34	45–50	R	Class 1	a	5	45	4	I
35	25–35	L	Class 2	a	6	80	9	I&II
36	35–40	L	Class 2	c	3	75	10	I&II
37	45–50	L	Class 2	a	3	70	7	I
38	45–50	R	Class 2	c	42	70	8	IV
39	45–50	L	Class 2	a	2	65	7	I
40	45–50	R	Class 2	c	4	60	10	I&II
41	45–50	R	Class 2	b	4	60	7	I
42	40–45	L	Class 2	a	3	90	7	I
43	40–45	L	Class 2	b	8	75	9	I&II
44	45–50	L	Class 3	b	1	90	6	I
45	50–55	R	Class 3	a	1	90	8	I&II
46	40–45	L	Class 4	a	4	110	10	I&II

*Abbreviation*: *a* Laparoscopic total hysterectomy, *b* Laparoscopic radical resection of cervical cancer, *c* Laparotomic cervical cancer resection procedures, *I* Replace D-J stents every 6–12 months, *II* Ureteroscopic endoureterotomy or re-expand with balloon every 6–12 months; *III* Removed the double-j stents after 3–6 months, *IV* Exchange nephrostomy tube every month

**Table 2:** Statistical of Results

	Successful operation		Failure operation	Total
	Removed D-J stents post operation	Replace D-J stents regularly		
Class 1	16	18	0	34
Class 2	0	8	1	9
Class 3	0	2	0	2
Class 4	0	1	0	1
Total	16	29	1	46
	45			

For Class 1, of the 34 patients who successfully underwent retrograde ureteric stenting,16 had their stents removed after 3–6 months. The other 18 needed to have stent replacement or have their endoureterotomy re-expanded every 6–12 months. The mean operating time was 35 min (range, 25–60 min), and the mean hospital stay was 3.8 days (range, 3–5 days).

Eleven Class 2, 3, and 4 patients underwent realignment of rigid and flexible ureteroscopy for stenting, needing replacement of their D-J stents or reexpanded with incision every 6 months. The antegrade flexible ureteroscopy ensured that the stents formed in the correct position and that the nephrostomy drainage was placed. The nephrostomy tube was left in place for 1–2 weeks to decrease intravesicular pressure and minimize reflux through stents. The other Class 2 patient, whose injury was healed by scar tissue, was diagnosed 42 days after the initial operation. After the ureteroscope was withdrawn, the scar’s “pseudo” tunnel narrowed again, requiring a repeat nephrostomy.She had to change the nephrostomy tube every month. The mean operating time was 80 ± 2 min (range, 60–110 min), and the mean hospital stay was 8.2 days (range, 6–10 days). To avoid sepsis and infection during hospitalization, the antibiotics should be used for 3 days routinely.

The catheter was indwelled in all 46 patients at the end of the operation and removed 2 weeks later. The overall catheterization success rate was 97.8%. There were no major complications, and blood loss was minimal. In the 6-month-to-3-year follow-ups (average, 18.6 months), ultrasound and intravenous pyelograms showed the ureter to be unobstructed, with the pelviureteric hydrocele significantly reduced or eradicated.

## Discussion

Almost all UVFs are linked to an iatrogenic lesion, which usually follows pelvic and gynecological surgery [7, 8]. It occurs as one of the rare and serious surgical complications [9]. The incidence of UVF can be attributed to the surgical technique [10] and its technical difficulty [11], although the risk factors for the underlying pathology of individual patients are not identical. Based on the surgical history, clinical symptoms, and auxiliary examination, diagnosis of UVF is not complicated [12].

For economic reasons, many surgeons dislike taking a patient who has undergone previous ureteral surgery, so UVF patients are often difficult to diagnose intraoperatively [13], with a median time to diagnosis of 3–30 days post injury. The basic tenet in treating a UVF is to restore the continuity of the urinary tract, protect kidney function, reduce localized stenosis, and avoid urinary fistula formation [14].

Due to secondary renal damage resulting from ureter-repair surgery or reconstruction, preoperative excretory urography or other auxiliary methods are needed to determine contralateral renal function, which is of great value in deciding how to deal with the damaged ureter. Urologists should adopt a comprehensive protocol according to the type, position, and degree of injuries.

Although the success rate, operative complications, and long-term outcome of traditional early surgical treatment for UVF are similar to delayed operation [15, 16], we suggest that UVF repair surgery or ureteric reimplantation be completed in the early stages [17, 18], which might avoid renal damage and reduce patient’s pain and financial burden. Generally, first-stage repair surgery should be available intraoperatively or within 24 h after ureteral injury. But for the delayed diagnosis cases and severe shockers with complicated injuries, repair surgery should be put off for 3–6 months after urine transfer [19].

In recent years, retrograde ureteric stenting has been recommended for the first stage of UVF, which reduces urine leakage in internal drainage and in inflammatory lesions [20]. UVF can typically be treated successfully by ureteric stenting as long as the ureter wall is continuous.

Generally speaking, there is no need to disconnect the ureter during transurethral ureteroscopy and retrograde stenting, UVF can typically be treated successfully by placing a D-J stent [21],which can replace the patient’s mental and physical trauma with easy acceptance, short hospitalization time, and fast recovery. Even if the UVF fails to heal, it can be used in first-stage treatment in full drainage to protect renal function and lay a good foundation for stage II surgery.

For some severe ureteric injuries, retrograde stenting is sometimes unsuccessful. Combining our practice with the literature review, we believe that the realignment retrograde/antegrade stenting approach is feasible by inserting the guide wire into the injury from the percutaneous nephroscope channel [22] and using the ureteroscope to grasp the guide wire out from the urethra, then pass the D-J stent along the guide wire and over the injury.

In a minority of situations, in which a patient presents with a severe ureteral injury—completely lacerated or atretic—such that a guide wire cannot be passed in either in a retrograde or antegrade fashion, endoscopic realignment for treatment and “cut-to-the-light” technique should be employed [6].

Compared with retrograde stenting, the extravasation of urine and washing fluid can be smoothly accomplished provided there is sufficient time and operating space for surgery. It would made stenting easier, more readily available, safer, and less invasive.

Therefore, it is appropriate for patients with a history of open pelvic and radical gynecologic surgery, multiple casualties, and those with severe fever, local inflammatory reaction, or severe shock.

Additional advantages of this method are as follows:

1. It is easy to find both ends of ureteral injury and approach its focus along the nephrostomy channel by means of double ureteroscopic realignment, which increases the success rate of prograde or retrograde ureteric stenting, avoiding additional surgery, or ureteric reimplantation or placement of a percutaneous nephrolithotomy (PCN) tube.
2. It ensures that the guide wire reaches the injury using direct vision and avoids exacerbating the ureter injury during adjustment of the guide wire’s position and direction during the procedure.
3. It is particularly appropriate for patients with failing retrograde ureteric stenting; patients who suffer from a severed ureter, severe injury, or ureter distortion; those who cannot tolerate long-term indwelling of a urinary catheter; and those with an advanced tumor.
4. The flank-reclining, split-leg position provides sufficient operating space to perform a PCN and retrograde ureteroscopy simultaneously.

Although the endoscopic realignment was successfully used, some obvious disadvantages of the method are as follows: In some cases with normal or mild hydronephrosis, it is difficult to gain access to a nondilated renal collecting system because of urine leakage.The ureter is easy to collapse and block after pulling out the ureteral stent in patients who have long suffered from defects of ureteric avulsion or transection (usually > 2 cm), serious stricture formation, repeated surgeries, or serious fibrosis of the ureteral surroundings. In addition, this procedure should be performed before the flexible ureteroscopy’s light no longer penetrates the tissue between the ureteroscopes. However, more studies with longer, consecutive follow-ups and mass cases are necessary to predict prognosis in the future.

## Conclusions

UVF is an uncommon iatrogenic complication of gynecologic surgery and difficult to diagnosis early. The incidence is more common in patients who have had radical hysterectomy. Minimally invasive methods using the D-J stent are safe and effective techniques for managing delayed UVF. We strongly recommend minimally invasive treatment of fistulae assisted with the ureteroscope to raise the cure rate, reduce the period of pain, and improve the survival rate.

## Abbreviations

D-J: Double J
PCN: Percutaneous nephroscopy
UVF: Uretero-vaginal fistula
